# Applications of machine learning for imaging-driven diagnosis of musculoskeletal malignancies—a scoping review

**DOI:** 10.1007/s00330-022-08981-3

**Published:** 2022-07-19

**Authors:** Florian Hinterwimmer, Sarah Consalvo, Jan Neumann, Daniel Rueckert, Rüdiger von Eisenhart-Rothe, Rainer Burgkart

**Affiliations:** 1 Department of Orthopaedics and Sports Orthopaedics, Klinikum rechts der Isar, Technical University of Munich, Munich, Germany; 2 Institute for AI and Informatics in Medicine, Technical University of Munich, Munich, Germany; 3 Department of Diagnostic and Interventional Radiology, Klinikum rechts der Isar, Technical University of Munich, Munich, Germany

**Keywords:** Primary musculoskeletal malignancies, Imaging-driven diagnosis, Diagnostic imaging, Machine learning, Deep learning

## Abstract

**Abstract:**

Musculoskeletal malignancies are a rare type of cancer. Consequently, sufficient imaging data for machine learning (ML) applications is difficult to obtain. The main purpose of this review was to investigate whether ML is already having an impact on imaging-driven diagnosis of musculoskeletal malignancies and what the respective reasons for this might be. A scoping review was conducted by a radiologist, an orthopaedic surgeon and a data scientist to identify suitable articles based on the PRISMA statement. Studies meeting the following criteria were included: primary malignant musculoskeletal tumours, machine/deep learning application, imaging data or data retrieved from images, human/preclinical, English language and original research. Initially, 480 articles were found and 38 met the eligibility criteria. Several continuous and discrete parameters related to publication, patient distribution, tumour specificities, ML methods, data and metrics were extracted from the final articles. For the synthesis, diagnosis-oriented studies were further examined by retrieving the number of patients and labels and metric scores. No significant correlations between metrics and mean number of samples were found. Several studies presented that ML could support imaging-driven diagnosis of musculoskeletal malignancies in distinct cases. However, data quality and quantity must be increased to achieve clinically relevant results. Compared to the experience of an expert radiologist, the studies used small datasets and mostly included only one type of data. Key to critical advancement of ML models for rare diseases such as musculoskeletal malignancies is a systematic, structured data collection and the establishment of (inter)national networks to obtain substantial datasets in the future.

**Key Points:**

*• Machine learning does not yet significantly impact imaging-driven diagnosis for musculoskeletal malignancies compared to other disciplines such as lung, breast or CNS cancer.*

*• Research in the area of musculoskeletal tumour imaging and machine learning is still very limited.*

*• Machine learning in musculoskeletal tumour imaging is impeded by insufficient availability of data and rarity of the disease.*

## Introduction

Malignant tumours of the musculoskeletal system represent a group of extraordinarily rare and heterogeneous tumour entities. For example, malignant bone tumours account for only about 0.2% of all human malignancies, but they occur more frequently in children (sixth most common cancer) and adolescents (third most common cancer) [1–3]. In addition to the pronounced rarity, the mostly unspecific history or clinical presentation also complicates early diagnosis and often leads to significant delays [3]. However, undelayed diagnosis is of paramount importance in musculoskeletal tumours, as the diagnostic window also has a direct impact on resectability and patient survival prognosis [2]. Thus, prompt referral to a specialised sarcoma centre is crucial when a malignant musculoskeletal tumour is suspected. However, delays of more than 12 months sometimes occur in clinical care reality, which can be explained not least by the fact that a general medical practitioner encounters only about three malignant musculoskeletal tumours in his/her professional life [4].

Especially the morphologic heterogeneity within musculoskeletal tumours complicates imaging entity or malignancy assessment and even limits the informative value of a biopsy. In sclerotic, blastic or cartilaginous lesions, as well as in tumours with a large necrotic area, retrieving adequate material from a biopsy is extremely challenging and requires a high degree of experience [5]. The rate of biopsy-related complications that adversely affect biopsy outcome or prognosis is reported to be 15–20%, with up to 12 times higher rates in non-specialist institutions [6]. Therefore, the importance of adequate diagnostic biopsy cannot be overstated in musculoskeletal tumours, which is why biopsy is considered the “first step of therapy” by many experts.

Image interpretation as a part of precision medicine plays an increasingly important role in the future of orthopaedic oncology, and novel, more comprehensive and specific analysis tools are urgently needed, especially for outpatient clinics with limited experience and resources for detection and interpretation of rare bone and soft tissue malignancies. Machine learning (ML) and the subset deep learning (DL) represent distinct applications of artificial intelligence (AI), which evolved from pattern recognition and learning theory. ML is just in its early stages in orthopaedics, and standardised approaches are not yet established. While complex data analysis of cancerous tissue through AI and imaging data is already widely applied for research purposes in some cancers (e.g. lung, breast or CNS cancer) [7], the application of these methods in orthopaedic oncology research is still very limited [8]. The fact that globally no far-reaching structures for systematic and structured data acquisition have yet been established (to the best of our knowledge) and that sarcomas are very rare and heterogeneous makes modern AI applications, for which a sufficient and qualitative amount of data is crucial, considerably more difficult. Although various methods for dealing with limited datasets have been developed (data augmentation [9], transfer learning [10], data simulation [11]), there is no way around building up appropriate structures and networks.

The main purpose of this review was to investigate whether ML can already substantially support image interpretation of musculoskeletal (MSK) malignancies with a focus on diagnostic tasks and what the respective reasons for this might be.

## Materials and methods

### Eligibility criteria

A scoping review of the literature was performed to identify ML applications in imaging of musculoskeletal malignancies based on the PRISMA statement [12]. Studies meeting the following criteria were included in this review:

- Primary malignant musculoskeletal tumours
- Application of machine learning or deep learning
- Imaging data or data retrieved from images
- Human or preclinical
- Written in English
- Original research articles

The following focus led to the exclusion of articles for this review:

- Metastases
- Histological data
- Secondary bone/soft tissue tumours
- Lymphoma
- Myeloma
- Benign, intermediate
- Review articles

Articles that contained benign or intermediate lesions but focused primarily on e.g. the detection of malignant lesions were included. In contrast, articles that did not contain data on malignant lesions were excluded. The focus was on malignant lesions because of their clinical relevance and difficulty in accurate assessment.

In December 2021, a thorough literature search through MEDLINE (PubMed), CENTRAL (Cochrane Library) and LISTA (EBSCO) was conducted. Grey literature was not considered. For the systematic search, the following search terms were used without any filters or limits:

- *((Artificial Intelligence) OR (Deep Learning) OR (Machine Learning)) AND (malignant) AND (tumour OR neoplasm OR cancer) AND (musculoskeletal OR sarcoma OR bone OR (soft tissue)) AND (imaging OR radiographic OR (computer-assisted) OR (image interpretation))*

Study titles were reviewed and evaluated by an MSK radiologist, an orthopaedic surgeon and a data scientist at our institution using the above selection criteria. All discrepancies were resolved by consensus. The results were summarised, and duplicates were discarded. All articles were initially screened for relevance by title and abstract to assess the inclusion criteria. The three authors independently performed a careful reading of the studies and extracted the data. The following information was extracted from each article: title, author, year of publication, tumour entity group, number of patients, malignancy, imaging modality, algorithm, model, task, applied metric, outcome label and if or if not focused on diagnosis. For the synthesis, studies with diagnosis-oriented tasks were further examined by retrieving the scores of the most common metrics and the number of class labels to assess the number of samples per class and illustrate a potential relationship between these parameters through linear analysis and a correlation coefficient. The level of evidence is level V.

### Statistical analysis

Continuous data is reported as mean with standard deviation (SD) or median with interquartile range (IQR), and the respective interval. Discrete data was reported as incidence and percentage share per entity. Due to the heterogeneous nature and the limited amount of data, a non-parametric test was chosen to calculate a correlation coefficient for metric values and number of samples per class label for the diagnosis-oriented studies.

## Results

### Selection and methodological characteristics

**Fig. 1:**
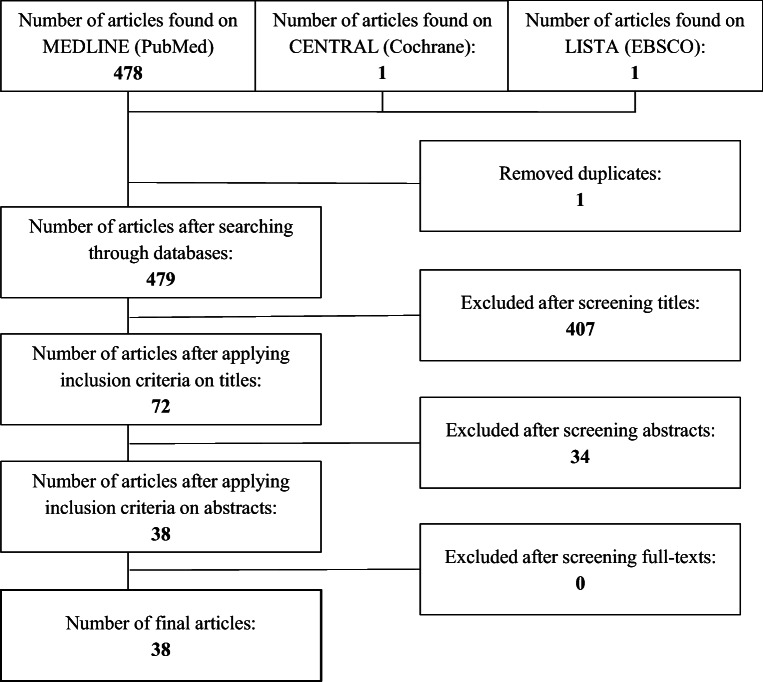
Selection process

The first search resulted in 480 references in the databases mentioned above. One duplicate was discarded and 38 articles subsequently met the eligibility criteria (Fig. 1) [8, 10, 13–51]. Table 1 displays the final selection of articles with authors and continuous and discrete parameters. Final articles were published between 1994 and 2021. All 38 articles addressed an application of ML or DL with imaging data of MSK malignancies. Three review articles were found and excluded from statistical analysis [8, 14, 25]. 75.7% (28) of the studies were conducted retrospectively, 8.1% (3) were conducted prospectively and 16.2% (6) did not clearly state the study design. 60.5% (23) of the studies focused on bone, while 39.5% (15) focused on soft tissue tumours. 50.3% of the cases included were from patients with benign tumours, 3.0% were from patients with intermediate tumours, 37.4% were from patients with malignant tumours, 5.4% were from patients with metastases, 3.6% were from patients without tumours (healthy) and 0.5% did not provide any information. Further details are reported in Tables 2 and 3.

**Table 1:** Final articles with continuous and discrete parameters. Acc and AUC values as well as number of labels were further investigated for articles with diagnosis-oriented tasks

Author	Year	Number of patients / cases	Healthy cases	Benign cases	Intermediate cases	Malignant cases	Metastases cases	Study design	Tumour entity group	Imaging modality	Radiomic data	Algorithm	Task	Model	Applied metric	Outcome label	Diagnosis-oriented	Acc	AUC	Number of labels
Bandyopadhyay et al	2019	150	0	0	0	150	0	Retrospective	Bone tumours	X-ray	No	Supervised	Classification	SVM, decision tree	acc, sens, Dice	Histopathological grading, staging	✓	0.85		2
Banerjee et al	2018	21	0	0	0	21	0	Retrospective	Soft tissue tumours	MRI	No	Supervised	Classification	AlexNet	acc, AUC, sens, spec	Tumour entities	✓	0.85		2
Chianca et al	2021	146	0	49	0	40	57	Retrospective	Bone tumours	MRI	Yes	Supervised	Classification	LogitBoost, SVM	AUC, sens, spec, acc	Malignancy	✓	0.90		2
Do et al	2021	1576	381	1061	0	134	0	Retrospective	Bone tumours	X-ray	No	Supervised	Classification, segmentation	UNet	acc, IoU	Segmented tumour, tumour entities	✓	0.99		3
Dufau et al	2019	69	0	0	0	69	0	Retrospective	Bone tumours	MRI	Yes	Supervised	Classification	SVM	AUC, sens, spec	Chemotherapy response assessment	**×**			
Eweje et al	2021	1060	0	582	0	478	0	Retrospective	Bone tumours	MRI	No	Supervised	Classification	Efficient-Net, logistic regression	acc, sens, spec, AUC	Malignancy	✓		0.79	2
Fields et al	2021	128	0	36	0	92	0	Retrospective	Soft tissue tumours	MRI	Yes	Supervised	Classification	Adaboost, random forest	AUC, sens, spec	Malignancy	✓		0.77	2
Gao et al	2021	30	0	0	0	30	0	Prospective	Soft tissue tumours	MRI	No	Supervised	Classification	VGG19	sens, spec, acc	Radiotherapy response assessment	**×**			
Gao et al	2020	30	0	0	0	30	0	Prospective	Soft tissue tumours	MRI	Yes	Supervised	Classification	SVM, logistic regression	AUC	Radiotherapy response assessment	**×**			
García-Gómez et al	2004	430	0	267	0	163	0	Retrospective	Soft tissue tumours	MRI	No	Supervised	Classification	K-nearest neighbour, SVM	sens, spec	Malignancy	✓	0.90		2
Gitto et al	2020	58	0	0	0	58	0	Retrospective	Bone tumours	MRI	Yes	Supervised	Classification	LogitBoost	acc, AUC	Histopathological grading	✓	0.75	0.78	2
Glass et al	1998	43	0	0	0	43	0	Retrospective	Bone tumours	MRI	No	Unsupervised	Segmentation	Neural network	acc, sens, spec	Chemotherapy response assessment	**×**			
He et al	2020	1356	0	679	0	360	317	Retrospective	Bone tumours	X-ray	No	Supervised	Classification	Efficient-Net	AUC, sens, spec, acc	Malignancy	✓	0.73		2
Holbrook et al	2020	79	0	0	0	79	0	Unknown	Soft tissue tumours	MRI	Yes	Supervised	Segmentation	SVM, neural network	Dice, AUC	Segmented tumour	**×**			
Hu et al	2021	160	0	90	0	70	0	Retrospective	Soft tissue tumours	MRI	Yes	Supervised	Classification	Least absolute shrinkage and selection operator	AUC, sens, spec, acc	Malignancy	✓	0.92	0.96	2
Hu et al	2014	141	0	71	0	70	0	Unknown	Bone tumours	X-ray	No	Supervised	Classification	SVM	acc, AUC, sens, spec	Tumour occurrence	✓	0.96		2
Huang et al	2020	12	0	0	0	12	0	Prospective	Bone tumours	MRI	No	Supervised	Classification	Random forest	AUC, sens, spec, acc	Chemotherapy response assessment	**×**			
Huang et al	2017	23	0	0	0	23	0	Unknown	Bone tumours	CT	No	Supervised	Segmentation	VGG16	Dice score	Segmented tumour	**×**			
Juntu et al	2010	135	0	86	0	49	0	Unknown	Soft tissue tumours	MRI	No	Supervised	Classification	SVM, neural network, decision tree	AUC, sens, spec, acc	Malignancy	✓	0.93		2
Leporq et al	2020	81	0	40	0	41	0	Retrospective	Soft tissue tumours	MRI	Yes	Supervised	Classification	SVM	AUC, sens, spec, acc	Malignancy	✓	0.95	0.96	2
Li et al	2019	210	0	154	0	56	0	Retrospective	Bone tumours	MRI	Yes	Supervised	Classification	SVM	AUC, sens, spec, acc	Tumour entities	✓		0.87	2
Liu et al	2021	643	0	392	93	158	0	Retrospective	Bone tumours	X-ray	No	Supervised	Classification	XGBoost, Inception V3	AUC, sens, spec, acc	Malignancy	✓		0.87	3
Pan et al	2021	796	0	412	169	215	0	Retrospective	Bone tumours	X-ray	No	Supervised	Classification	Random forest	AUC, acc	Malignancy	✓	0.95	0.97	3
Peeken et al	2019	221	0	221	0	0	0	Retrospective	Soft tissue tumours	CT	Yes	Supervised	Classification	Random forest	AUC, Dice	Histopathological grading	✓		0.64	2
Peeken et al	2018	136	0	0	0	136	0	Retrospective	Soft tissue tumours	MRI, CT	No	Supervised	Classification	Random forest	AUC, sens, spec, acc	Prognosis	**×**			
Reinus et al	1994	709	0	492	0	217	0	Retrospective	Bone tumours	X-ray	No	Supervised	Classification	Neural network	acc	Malignancy	✓	0.85		2
Shen et al	2018	36	0	15	0	21	0	Unknown	Bone tumours	X-ray	No	Supervised	Classification	Random forest, SVM	AUC, sens, spec, acc	Malignancy	✓	0.85	0.94	2
Terunuma et al	2018	1	N/A	N/A	N/A	N/A	N/A	Retrospective	Bone tumours	X-ray	No	Supervised	Object detection, segmentation	SegNet	Jaccard index	Segmented tumour	**×**			
von Schacky et al	2021	934	0	623	0	311	0	Retrospective	Bone tumours	X-ray	No	Supervised	Object detection, segmentation, classification	Mask-RCNN	acc, sens, spec, IoU, Dice	Malignancy	**×**			
Vos et al	2019	116	0	58	0	58	0	Retrospective	Soft tissue tumours	MRI	Yes	Supervised	Classification	SVM, random forest	AUC, sens, spec	Tumour entities	✓		0.89	2
Wang et al	2021	227	0	147	0	80	0	Retrospective	Bone tumours	US	No	Supervised	Classification	VGG16	acc, sens, spec, AUC	Malignancy	✓	0.79	0.91	2
Wang et al	2020	206	0	105	0	93	8	Retrospective	Soft tissue tumours	MRI	Yes	Supervised	Classification	SVM, generalised linear models, random forest	AUC, sens, spec, acc	Malignancy	✓	0.86	0.92	2
Yin et al	2019	120	0	0	30	54	36	Retrospective	Bone tumours	MRI	Yes	Supervised	Classification	Random forest	AUC, acc	Segmented tumour, tumour entities	✓	0.71	0.77	3
Yin et al	2019	95	0	0	42	53	0	Retrospective	Bone tumours	CT	Yes	Supervised	Classification	Random forest	acc, AUC	Tumour entities	✓	0.90	0.98	2
Yin et al	2021	795	0	215	0	399	181	Retrospective	Bone tumours	CT	Yes	Supervised	Classification	Random forest	AUC, acc	Tumour entities	✓	0.88	0.93	2
Zhang et al	2020	51	N/A	N/A	N/A	N/A	N/A	Retrospective	Soft tissue tumours	MRI, CT	No	Supervised	Classification	Inception-v3	acc, AUC	Histopathological grading	✓	0.86	0.97	3
Zhang et al	2019	35	0	0	0	35	0	Retrospective	Soft tissue tumours	MRI	Yes	Supervised	Classification	Random forest, SVM	AUC, sens, spec, acc	Histopathological grading	✓	0.88	0.92	2
Zhang et al	2018	23	0	0	0	23	0	Unknown	Bone tumours	CT	No	Supervised	Segmentation	ResNet-50	Dice, sens	Segmented tumour	**×**			

*SVM* support vector machine, *IoU* intersection over union *N/A* not assessed

**Table 2:** Continuous parameters with interval, median, mean IQR, and standard deviation

Continuous parameters					
Parameter	Interval	Median	IQR	Mean	Std
Year of publication	[1994; 2021]	2020	3	2018	6
Number of patients/cases	[1; 1565]	132.0	180.5	292.0	392.0
Healthy	[0; 381]	0.0	0.0	10.6	62.6
Benign	[0; 1061]	38.0	154.2	154.8	248.3
Intermediate	[0; 169]	0.0	4.6	9.3	32.0
Malignant	[12; 478]	69.5	79.5	115.1	113.4
Metastases	[0; 317]	0.0	4.3	17.1	60.4

*IQR* interquartile range, *std* standard deviation

**Table 3:** Discrete parameters with incidence and percentage share per entity

Discrete parameters			
Parameter	Entity	Σ	%
Study design			
	Retrospective	28	75.7%
	Prospective	3	8.1%
	Unknown	6	16.2%
Task			
	Classification	33	80.5%
	Segmentation	6	14.6%
	Object detection	2	4.9%
Model			
	AlexNet	1	1.9%
	LogitBoost	2	3.8%
	Support vector machine	14	26.4%
	U-Net	1	1.9%
	Efficient-Net	2	3.8%
	Logistic regression	2	3.8%
	Adaboost	1	1.9%
	Random forests	12	22.6%
	VGG19	1	1.9%
	k-nearest neighbour	1	1.9%
	Neural network	4	7.5%
	LASSO	1	1.9%
	VGG16	2	3.8%
	Decision tree	2	3.8%
	XGBoost	1	1.9%
	Inception v3	2	3.8%
	SegNet	1	1.9%
	Mask RCNN	1	1.9%
	Generalised linear model	1	1.9%
	ResNet-50	1	1.9%
Diagnosis oriented			
	Yes	27	71.1%
	No	11	28.9%
Outcome label			
	Segmented tumour	6	14.6%
	Tumour entities	7	17.1%
	Tumour occurrence	1	2.4%
	Histopathological grading	5	12.2%
	Radiotherapy response	2	4.9%
	Chemotherapy response	3	7.3%
	Malignancy	15	36.6%
	Staging	1	2.4%
	Prognosis	1	2.4%
Tumour group			
	Bone tumour	23	60.5%
	Soft tissue tumour	15	39.5%
Imaging modality			
	MRI	22	55.0%
	CT	7	17.5%
	X-ray	10	25.0%
	US	1	2.5%
Radiomic data			
	Yes	16	42.1%
	No	22	57.9%
Algorithm			
	Supervised	37	97.4%
	Unsupervised	1	2.6%
	Reinforcement	0	0.0%
Applied metric			
	Accuracy	29	25.4%
	Sensitivity	25	21.9%
	Specificity	23	20.2%
	AUC	28	24.6%
	Jaccard index	1	0.9%
	Intersection over union	2	1.8%
	Dice score	6	5.3%

*LASSO* Least Absolute Shrinkage and Selection Operator

### Narrative review of best studies

Several studies have presented novel and interesting implementations. However, we would like to highlight two studies that, in our opinion, provide very intriguing frameworks. Liu et al [35] demonstrated a ML-DL fusion model that integrates not only imaging but also clinical data to assess the malignancy of tumours. This approach is similar to the diagnostic procedure a radiologist would use to diagnose MSK lesions. A second noticeable study was published by von Schacky et al [42]: they presented a multi-task DL model that shows the potential of state-of-the-art DL by simultaneously detecting, segmenting and classifying image data. To classify the DL results in the context of “man vs. machine,” they were also compared with the results of radiologists of different experience levels demonstrating strengths and limitations of DL with limited data.

### In-depth investigation of diagnosis-oriented tasks

Twenty-seven (71.1%) of the studies were diagnosis-oriented and mainly aimed at classification tasks [10, 13, 15, 16, 18, 19, 22, 23, 26, 28, 29, 32–37, 39, 40, 43–49, 51]. A median accuracy (Acc) of 0.88 with an interval of [0.71; 0.99] was found. For the area under the curve (AUC), the median resulted in 0.92 with a corresponding interval of [0.64; 0.98]. For the number of labels, a median of 2 with an interval of [2;3] was found. Further details are shown in Table 4.

**Table 4:** Continuous parameters of diagnosis-oriented studies with interval, median, mean and standard deviation

Continuous parameters of diagnosis-oriented parameters					
Parameter	Interval	Median	IQR	Mean	std
ACC	[0.71; 0.99]	0.88	0.07	0.87	0.07
AUC	[0.64; 0.98]	0.92	0.14	0.88	0.09
Number of labels	[2; 3]	2	0	2.19	0.39

*IQR* interquartile range, *std* standard deviation

**Fig. 2:**
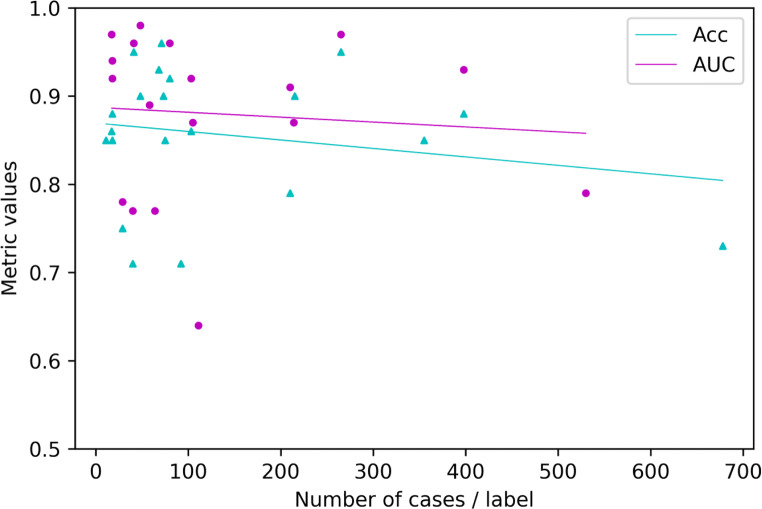
Distribution of final metric scores against the mean number of samples per class label

Figure 2 demonstrates the findings of a linear analysis of the metric values Acc and AUC on the vertical axis and the quotient of total number of cases and number of labels per class (= mean number of samples per class). Further, a correlation coefficient for each metric and the mean number of samples per class was calculated. The number of studies examined is limited, and the data found show considerable heterogeneity. Subsequently, a Spearman’s rank-order correlation coefficient, which is a measure for linear correlation between two datasets and does not assume that both datasets are normally distributed, was applied. We chose |*ρ*| > 0.5 to infer a significant direct or indirect correlation between two parameters for this study. The correlation coefficient for Acc and AUC against the mean number of samples per class resulted in *ρ* = − 0.204 / *ρ* = − 0.153, respectively. Therefore, both results represent no significant correlation coefficient.

## Discussion

The most important finding of the presented review was that imaging-driven diagnosis for MSK malignancies does not yet experience significant impact by ML applications and this has several reasons associated with data.

The main issue might be the availability of data. In most research institutes, a systematic and structured collection of quality data does not yet seem to take place or has only recently been introduced. This can be derived from the fact that datasets in general are comparably small and dataset size is not increasing yet. Consequently, even if according patient data is existing, this does not necessarily imply data is present in a format, validity, accessibility, consistency and completeness feasible for data science. In addition, sarcomas are a very rare entity of cancer, which does not allow for fast gathering of sufficient prospective data. Terenuma et al [41] developed a technique to obtain multiple images from a single patient, which is from a data science perspective very intriguing, but does not provide enough data for a clinical application and is not generally transferable to any other study. Several mathematical techniques to cope with limited data have emerged (e.g. transfer learning [10], data augmentation [9]). However, these techniques can at this point only support an AI task, but not solve the issue of limited data. For rare diseases, building networks and databases on a national or even international basis might be a future solution. Another reason might be the considerably limited amount of research in the field of orthopaedic oncology, which can again partly be explained by insufficient data. With the respectively adapted search term, more than 1300 articles can be found for lung malignancies and even more than 2200 articles for breast malignancies, while only 480 articles were detected for MSK malignancies (initial search, each in December 2021). ML in general is still in its infancy, but more so in MSK and orthopaedic oncology.

A further finding was presented by synthesising the relationship of number of cases and number of labels per class against the metric values. In the research field of AI, it is common knowledge that the amount of data has profound impact on the model performance [10, 11, 52]. Nonetheless, Fig. 2 tells a different story. The median number of samples per class resulted in 75 and 59.3% of the diagnosis-oriented studies had less than 100 samples per class. Further, the mean metric scores of studies with fewer than 100 samples per class (Acc 0.86, AUC 0.89) were slightly higher than those of studies with more than 100 samples per class (Acc 0.85, AUC 0.86), as indicated by the linear regression lines in Fig. 2. This would suggest that less data leads to higher results. One explanation for these unexpected results could be the class imbalance: several studies developed models to classify tumour malignancy, for example [15, 18, 19, 22, 26, 28, 32, 33, 35, 36, 39, 40, 44, 45]. Benign MSK tumours occur more often than malignant MSK tumours, which results in a class imbalance in the dataset. Such an imbalance can lead to spuriously high metric values, especially for AUC. A detailed and interdisciplinary interpretation of results with regard to composition of data is crucial. Another issue associated with limited datasets and class imbalance is that specific classes of data might be sparse. Therefore, overfitting may occur, resulting in suboptimal results.

Yet another indication is that problem statements of most studies do not reflect real clinical scenarios. Most studies aim at distinguishing two to three specific tumour entities [10, 16, 34, 43, 46–48] or assessing tumour malignancy [15, 18, 19, 22, 26, 28, 32, 33, 35, 36, 39, 40, 42, 44, 45]. If one fed a third entity to a two-entity classifier, the model would try to fit the third entity into one of the first two entity classes. While confining a tumour entity from another is an imperative step in tumour assessment, nonetheless, most sarcoma diagnoses are incidental findings, and in daily practice, MSK radiologists and orthopaedic surgeons are first confronted with detecting a potential sarcoma at all [1, 4, 53]. Whereas von Schacky et al [42] aimed at differentiating various tumour entities, thus modelling a more realistic clinical scenario, the results were only moderate. More general models are needed to comply with clinical needs and difficulties. However, we hypothesise that this is again very difficult to achieve due to the very limited amount of data available and probably also closely related to the distribution of the data. Naturally, the quality and problems of AI models cannot be assessed by dataset size and data distribution alone, but data undoubtedly have major impact on the overall performance and clinical relevance.

### No biopsy-focused studies

The most applied outcome labels among the 38 investigated original research articles were tumour malignancy (15, 36.6%) [15, 18, 19, 22, 26, 28, 32, 33, 35, 36, 39, 40, 42, 44, 45], tumour entities (7, 17.1%) [10, 16, 34, 43, 46–48] and segmented tumour (6, 14.6%) [16, 27, 31, 41, 46, 50]. A distinct finding of this review is that although a biopsy is a crucial step in the diagnostic process of MSK malignancies, there is no study focused on radiological images and biopsies. Retrieving relevant biopsy material—for example, via CT-guided needle biopsy—is a highly complex task and requires significant experience. From this, it could be derived that ML research in the field of MSK malignancies is currently not mainly oriented on medical needs, but models and research questions are built around available data. This underlines that ML is still in its very infancy in MSK tumour research.

### MRI and radiomics

MRI is the most popular kind of imaging data for ML analysis at this point (55.0%, 22). This might be explained by the fact that MR imaging plays a fundamental role in the assessment of sarcomas due to superior soft tissue contrast and the desire to reduce unnecessary radiation dose. But also, from a data science perspective, this is comprehensible: with one patient, multiple 2D data samples (or one 3D data sample) are produced. Additionally, various image planes and weightings are possible. This suggests that less patients are necessary to acquire more data.

Likewise, radiomics appears to be on demand. 42.1% of articles (16) utilised radiomic data [15, 17, 19, 21, 23, 27, 28, 33, 34, 37, 43, 45–48, 51], while only 17.5% (7) integrated CT, 25.0% (10) X-ray and 2.5% (1) US. With radiomics, a large number of quantitative features can be extracted from imaging data. These are combined with other patient data and can be mined with modern techniques of e.g. bioinformatics and data science. In consequence, the popularity of radiomics might be associated with the capability to extract additional information from images and therefore tackle the issue of small datasets.

### Limitations

This review article has several limitations. The major limitation is the early stage of the examined studies. Because ML in orthopaedic oncology is still in its infancy, most studies are also at an early stage, making it difficult to examine the impact of the studies presented and assess their quality. Most studies were not published until 2021. Further, the mean number of cases per study is 292. While a limited number of cases is related to the type of entities studied [53], the number is very small in the context of ML applications. These facts underline the early stage of the studies. Another limitation is the overall heterogeneity of the examined studies. We restricted the tumour entities and the type of data by the eligibility criteria. However, we did not impose any restrictions on ML algorithms, models, or tasks. Thus, the studies presented three distinct algorithm types, 20 different models and nine groups of outcome labels for various tasks.

## Conclusion

In conclusion, for a rare disease, there are very limited amounts of data and no established large-scale networks between multiple national and international facilities yet. The impact of imaging-driven ML research in other disciplines is already present [52]. Also, several studies presented in this review demonstrated that ML can selectively support imaging-driven diagnosis for MSK malignancies. However, until statistically robust results can be achieved and clinically relevant models to cope with heterogeneous cases an orthopaedic surgeon or MSK radiologist encounters on a regular basis can be developed, data quality and quantity have to be improved. An expert radiologist from a specialised centre has seen thousands of images in his/her professional life and incorporates meta data as well as other factors into his/her decision-making process. In contrast, the presented studies only worked with 1 [41] up to 1576 [16] cases mostly focusing on one single kind of data and imaging modality.

The key to bring ML to a level where it can substantially impact clinical image interpretation in the diagnosis of MSK malignancies is data: establishing national and international networks, implementing a systematic and structural data acquisition and finally integrating multimodal data comparable to expert radiologists.

## Abbreviations

Acc: Accuracy
AI: Artificial intelligence
AUC: Area under the curve
DL: Deep learning
IoU: Intersection over union
IQR: Interquartile range
ML: Machine learning
MSK: Musculoskeletal
SD: Standard deviation
SVM: Support vector machine

## Declarations

### Guarantor

The scientific guarantor of this publication is Prof. MD Rainer Burgkart.

### Statistics and biometry

One of the authors has significant statistical expertise (Florian Hinterwimmer).

### Methodology

• retrospective

• performed at one institution
